# Eosinophilic Oesophagitis in Adults: National Centre Retrospective Study in an Irish Cohort

**DOI:** 10.1111/cea.70016

**Published:** 2025-02-19

**Authors:** Olga Fagan, Ciaran Judge, Ciara Ryan, Niall Conlon, Claire L. Donohoe, Joanne C. Masterson, Susan Mc Kiernan

**Affiliations:** ^1^ St. James's Hospital Trinity College Dublin Dublin Ireland; ^2^ Allergy Inflammation Remodeling Research Laboratory, Kathleen Lonsdale Institute for Human Health Research, Department of Biology National University of Ireland, Maynooth Co. Kildare Ireland

**Keywords:** clinical‐remission, EoE, eosinophilic oesophagitis, fibrostenotic EoE, histological‐remission

**Summary**

- Real‐world oesophageal biopsy approaches are often suboptimal and may contribute to delayed diagnosis.
- EoE may not be as rare in Irish adults as previously thought.

**Abbreviations**
BSG: British Society for Gastroenterology
EoE: eosinophilic oesophagitis
EREFS: endoscopic reference score
FP: fluticasone proprionate
GI: gastrointestinal
hpf: high‐power field
OGD: oesophagogastroduodenoscopy
OVB: oral viscous budesonide
PPI: proton pump inhibitor
SD: standard deviation
SJH: St James's Hospital

To the editor,

Incidence and prevalence of eosinophilic oesophagitis (EoE) may be increasing. Patients with fibrostenotic disease often present with dysphagia, food bolus obstruction and the inflammatory‐predominant phenotype with vomiting and reflux [1]. Delayed diagnosis and treatment correlate with fibrosis [2]. Improved physician awareness could enable more timely diagnosis, reduce disease progression, and thus associated complications, treatment refractory disease and poorer quality of life. An Irish 2011 study indicated EoE was rare, noting an increasing referral pattern [3].

A retrospective study of St James's Hospital (SJH) pathology records (January 2016–December 2020) for patients with recorded eosinophilia on oesophageal biopsy was conducted. Those meeting the EoE [4] criteria were included. The incidence, characteristics and management of EoE patients were reviewed, when no specialty clinic existed and patients were managed by multiple disciplines. This clinical audit study was SJH Research and Innovation (7989) approved; ethics review was not required.

EoE endoscopic phenotypes representing severity of inflammation versus remodelling included (i) inflammatory (oedema, furrows and exudate), (ii) fibrostenotic (rings and strictures) and (iii) mixed (i+ii combined) [1]. Remission was reviewed separately across clinical, endoscopic and histologic criteria. ‘Clinical‐remission’ refers to complete resolution of symptoms, whereas ‘clinical‐response’ was any improvement. A single operator reviewed endoscopic photographs and calculated EREFS. ‘Endoscopic‐remission’ was an EREFS of 0 and ‘endoscopic‐response’ as any improvement. ‘Histological‐remission’ was < 15‐eosinophils/hpf and ‘histological‐response’ as any improvement in eosinophils/hpf. Other histological features of EoE were not routinely reported.

New EoE patients were identified each year, divided by the Dublin City region adult population (aged ≥ 15 years) at the time of study, yielding annual incidence rate (cases/100,000‐population). Cumulative prevalence was calculated similarly. Annual incidence of adult EoE diagnosis at SJH remained stable, ranging between 1.1 (2015) and 2.6 (2020); prevalence increased steadily, to a cumulative 12.2 by 2020.

A total of 133‐patients with biopsy eosinophilia were identified; of these, 78 met the EoE diagnostic criteria (including ≥ 15eos/hpf) [4]. Symptoms included: dysphagia (55%), reflux (8%), impacted food bolus (7%), food sticking (5%), chest pain (1%) and no indication (20%) was documented on index OGDs, with no significant difference among phenotypes.

Mean age at OGD/biopsy was 33‐years (SD14); males predominated (*n* = 61 [78%]). Mean outpatient follow‐up was 46‐months. Duration of pre‐diagnosis symptoms was longer in phenotypes with fibrosis (mixed = 6‐years; fibrostenotic = 7‐years) versus inflammation (3.4‐years; *p* = 0.0316).

Endoscopic phenotype data were available for 75/78‐patients. Evaluating the index OGD, the median index EREFS score was 3 and did not improve on follow‐up; phenotypes were inflammatory *n* = 34 (45%), mixed *n* = 34 (45%) and fibrostenotic *n* = 7 (10%). Only 35% had adequate oesophageal biopsies taken for diagnosis [5], with adequate biopsy rates in 2016 = 24%, 2017–2018 = 38% and 2019–2020 = 35% and time to diagnosis of ~6‐years (2016–2020).

Of the 248 procedures, *n* = 54 (22%) involved therapeutic interventions (balloon dilatation *n* = 35 [63%]; food bolus extraction *n* = 15 [28%]; bougie dilatation *n* = 5[9%]). More therapeutic procedures were performed in fibrostenotic and mixed phenotypes (86% and 47%, respectively) versus the inflammatory group (6%; *p* = 0.0001). Thirty‐three percent (*n* = 26/78) underwent therapeutic procedure(s) on their index OGD (*n* = 124/248), that is, 49% of all OGDs. These patients underwent 4.8 OGDs versus 2.4 OGDs for those not requiring intervention (*p* = 0.00054).

Fifty‐eight of 78‐patients had adequate clinical follow‐up data. Of these, *n* = 51 (88%) achieved some symptom improvement, but only *n* = 20 (35%) achieved full clinical‐remission, leaving *n* = 7 (12%) with no improvement.

Stricture presence (7‐v‐0 patients; *p* = 0.029) and longer symptom duration (6.1‐v‐3.4 years; *p* = 0.0316) were significantly different in those not achieving clinical‐remission (Figure 1). Those achieving clinical‐remission (*n* = 20/58) did so with OVB‐alone *n* = 6 (30%), FP+PPI *n* = 4 (20%), PPI‐alone *n* = 4 (20%), FP‐alone *n* = 3 (15%), OVB+PPI *n* = 2 (10%) and dairy exclusion *n* = 1 (5%). Where clinical‐remission had adequate follow‐up (*n* = 13), *n* = 10 (77%) achieved histological‐remission and no significant age or sex difference was noted.

**FIGURE 1:**
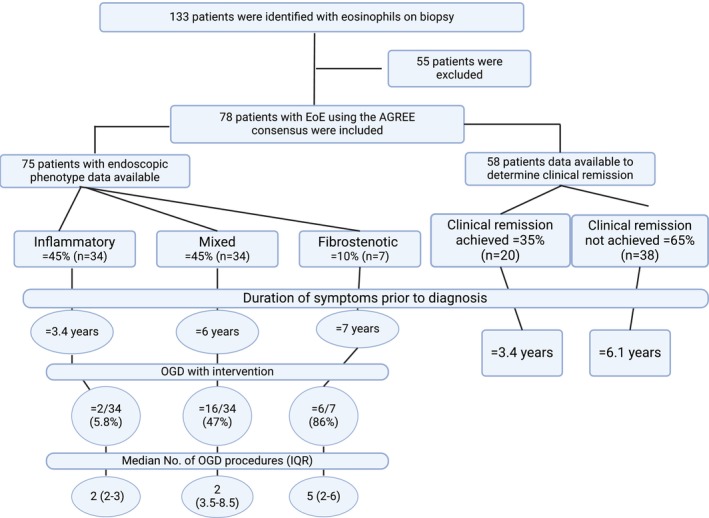
Flow diagram of inclusion and exclusion criteria, endoscopic phenotypic data and factors associated with clinical‐remission (AGREE consensus 2018 Dellon, IQR interquartile range, OGD oesophagogastroduodenoscopy).

Of the 57‐patients exposed to PPI‐therapy, at last follow‐up *n* = 11 (< 20%) remained on sole PPI‐therapy, 3 were lost to follow‐up; and of the 8 remaining (mean follow‐up 4 years), 5 remained on therapy. Of those with endoscopic follow‐up, 4/5 demonstrated endoscopic‐remission and 3/5 remained in histological‐remission. Often, PPI‐response was not routinely assessed with follow‐up endoscopy and patients who did not improve symptomatically were escalated to topical corticosteroids.

Among steroid treated patients with follow‐ups reported, 36/44 (82%) demonstrated symptomatic improvement (39% clinical‐remission, 43% clinical‐response); *n* = 20/26 (77%) demonstrated endoscopic improvement (38.5% remission, 38.5% response); and 21/28 (75%) demonstrated histological improvement (46% remission, 29% response). Poorer rates of all responses occurred in FP‐versus‐OVB‐treated patients. Compliance was not assessed. Significantly fewer fibrostenotic group patients were exposed to steroid‐therapy compared with the overall cohort (67%‐v‐80% *p* = 0.023*).

Although this study is limited by its small sample size, retrospective nature, tertiary centre location, with missing data (e.g., standard reporting of histology features), it is the first detailed Irish study since 2011 and provides insight into the natural history of disease. A prospective study is required. Versus similar centres, our patient cohort demonstrated poor clinical/endoscopic and histologic‐remission rates and a more severe phenotype with higher EREFS's, which did not improve in follow‐up. Although our incidence of oesophageal stricturing disease agrees with literature [2, 6], such patients required multiple endoscopic interventions. Longer duration of symptoms prior to diagnosis was associated with fibrosis, poorer clinical remission rates and increased requirement of endoscopic intervention thus impacting cost and quality of life. Other contributory factors may include multifactorial refractoriness, suboptimal biopsy strategies which may negatively influence time to diagnosis, poor follow‐up rates underpinned by variability in care due to absence of a dedicated clinic. Lastly, access in Ireland to approved medication is challenging. Off‐label topical steroid treatments can be cumbersome and lead to compliance issues, which may influence observed stasis in clinical‐remission rates and follow‐up EREFS's. The end result of these factors is delayed diagnosis and lack of consistent effective treatment resulting in increased inflammation; this may account for a more severe phenotype. A dedicated clinic has since been established aimed at standardising and improving care. Improved patient outcomes and quality of life could result from improvements in (i) primary care education, (ii) access to specialist services and medications and (iii) improved outpatient follow‐up.

## Author Contributions

Study concept and design: Olga Fagan, Ciaran Judge, Niall Conlon, Claire L Donohoe, Joanne C. Masterson and Susan Mc Kiernan. acquisition of data: Olga Fagan, Ciaran Judge amd Ciara Ryan. Analysis and interpretation of data: Olga Fagan, Ciaran Judge, Joanne C. Masterson and Susan Mc Kiernan. Drafting of the manuscript: Olga Fagan and Joanne C. Masterson. Critical revision of the manuscript for important intellectual content: Olga Fagan, Ciaran Judge, Ciara Ryan, Niall Conlon, Claire L Donohoe, Joanne C. Masterson and Susan Mc Kiernan.

## Conflicts of Interest

The authors declare no conflicts of interest.

## Data Availability

The data that support the findings of this study are available from the corresponding author upon reasonable request.
